# Patterns of long-term survival following Ipilimumab (Ipi): the Memorial Sloan Kettering Cancer Center 10-year metastatic melanoma (MM) experience

**DOI:** 10.1186/2051-1426-2-S3-P117

**Published:** 2014-11-06

**Authors:** David B Page, Jarushka Naidoo, Parisa Momtaz, Kita Bogatch, Deborah Kuk, Katherine Panageas, Jianda Yuan, Jedd D Wolchok, Michael Postow

**Affiliations:** 1 Memorial Sloan Kettering Cancer Center, New York, NY, USA; 2 Immune Monitoring Facility, Sloan Kettering Institute, New York, NY, USA

## Background

In two Phase III randomized trials in MM, Ipi improved median overall survival (OS) [1,2]. Here, we evaluate OS and characterize post-Ipi treatment patterns among long-term survivors from a single-institution cohort of patients (pts) treated with Ipi.

## Methods

Through a search of institutional databases, we identified 766 pts with MM treated with Ipi between 1/1/2003 and 12/31/2013. As of 4/1/2014, 96 pts have survived ≥2 yrs, measured from first dose of Ipi. OS was calculated utilizing the Kaplan-Meier method. Disease control was defined as the duration from initiation of Tx until initiation of subsequent systemic Tx or death.

## Results

With a median follow-up of 17mo (range 0-9yr), the median OS for the entire cohort of 766 pts was 15mo, with a 2-yr OS of 41%. Of the 80 pts with OS ≥2 yrs post-Ipi for whom data are available, 75% (n = 60/80) remain alive and 30% (n = 24/80) remain progression-free following Ipi, with median Ipi disease control of 15mo (range: 3 to 107+mo). Among pts with progression (n = 56), 57% exhibited disseminated progression, 29% oligometastatic progression, and 15% CNS-only progression. The most frequent Tx at first progression was locoregional (n = 29), employed at a median of 11mo post-Ipi (range: 3 to 55mo) and associated with median 15+mos disease control (range 1 to 73+mo) (table 1). In pts requiring post-Ipi systemic Tx, long-term disease control was observed across multiple systemic Tx's (table 2).

**Table 1:** Pts receiving locoregional Tx at first progression.

Locoregional Tx	# pts	# pts achieving ≥1yr disease control
**CNS, surgery and/or RT**	9/80 (11%)	5/9 (56%)
**Non-CNS, surgery**	11/80 (14%)	8/11 (73%)
**Non-CNS, RT**	6/80 (8%)	3/6 (50%)
**Ablation**	2/80 (3%)	1/2 (50%)

**Table 2:** Post-Ipi Systemic Txs.

Post-Ipi Systemic Tx	# pts	# pts achieving ≥1yr disease control*
**Cytotoxic Therapy**	11/80 (14%)	5/10 (50%)
**Anti-PD-1/PD-L1**	16/80 (20%)	11/15 (73%)
**BRAF inhibitor**	10/80 (13%)	7/9 (78%)
**Ipi re-induction**	18/80 (23%)	5/16 (31%)
**Other clinical trial**	13/80 (16%)	1/13 (8%)

*pts with ongoing disease control and <1yr follow up excluded

## Conclusion

Within this single-institution cohort, the median OS and 2-yr OS were greater than reported previously in Phase III trials [1,2]. Potential reasons for this survival advantage include: referral bias, heterogeneous Ipi dosing/schedule, and access to subsequent trials (i.e. anti-PD-1/PD-L1, BRAF inhibitor). The majority of long-term survivors required subsequent Tx, however prolonged disease control was achieved with a range of Tx's. Pts who experience oligometastatic/CNS-only progression following Ipi may achieve prolonged disease control with locoregional Tx alone.
